# Residual-based approach for authenticating pattern of multi-style diacritical Arabic texts

**DOI:** 10.1371/journal.pone.0198284

**Published:** 2018-06-20

**Authors:** Saqib Hakak, Amirrudin Kamsin, Shivakumara Palaiahnakote, Omar Tayan, Mohd. Yamani Idna Idris, Khir Zuhaili Abukhir

**Affiliations:** 1 Faculty of Computer Science and Information Technology, University of Malaya, Kuala Lumpur, Malaysia; 2 Faculty of Computer Science, Taibah University, Madinah, Saudi Arabia; 3 Academy of Islamic Studies, University of Malaya, Kuala Lumpur, Malaysia; King Saud University, SAUDI ARABIA

## Abstract

Arabic script is highly sensitive to changes in meaning with respect to the accurate arrangement of diacritics and other related symbols. The most sensitive Arabic text available online is the Digital Qur’an, the sacred book of Revelation in Islam that all Muslims including non-Arabs recite as part of their worship. Due to the different characteristics of the Arabic letters like diacritics (punctuation symbols), *kashida* (extended letters) and other symbols, it is written and available in different styles like *Kufi*, *Naskh*, *Thuluth*, *Uthmani*, etc. As social media has become part of our daily life, posting downloaded Qur’anic verses from the web is common. This leads to the problem of authenticating the selected Qur’anic passages available in different styles. This paper presents a residual approach for authenticating *Uthmani* and plain Qur’an verses using one common database. Residual (difference) is obtained by analyzing the differences between *Uthmani* and plain Quranic styles using XOR operation. Based on predefined data, the proposed approach converts *Uthmani* text into plain text. Furthermore, we propose to use the Tuned BM algorithm (BMT) exact pattern matching algorithm to verify the substituted *Uthmani* verse with a given database of plain Qur’anic style. Experimental results show that the proposed approach is useful and effective in authenticating multi-style texts of the Qur’an with 87.1% accuracy.

## I. Introduction

**Fig 1:**
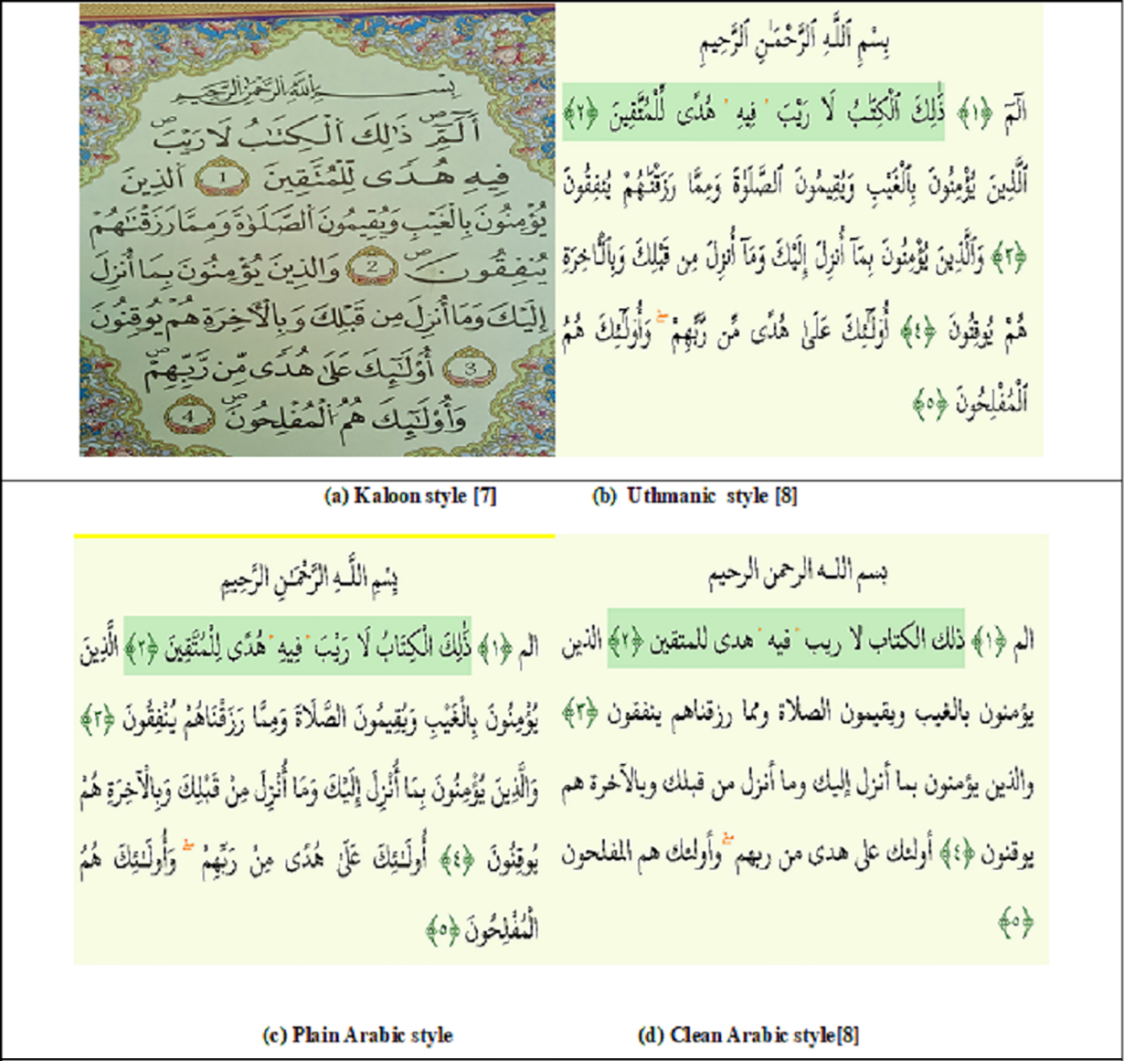
Different writing styles of Digital Holy Quran [8].

Digital versions of the Quran are made available online in different styles for reading purposes. Although the trend of reading digitized online versions of the Quran is increasing, the issue of credibility and authenticity is drawing more and more public attention [1–3]. Since the Quran is a sensitive script, its authentication and integrity are of greatest concern [1, 4–6]. The Quran is written in Arabic language and in different styles such as plain text (mostly used in countries like India, Pakistan and Bangladesh), Uthmanic, Kufi, Kaloon and other such styles [7–8]. Several such styles are shown in Fig 1.

**Fig 2:**
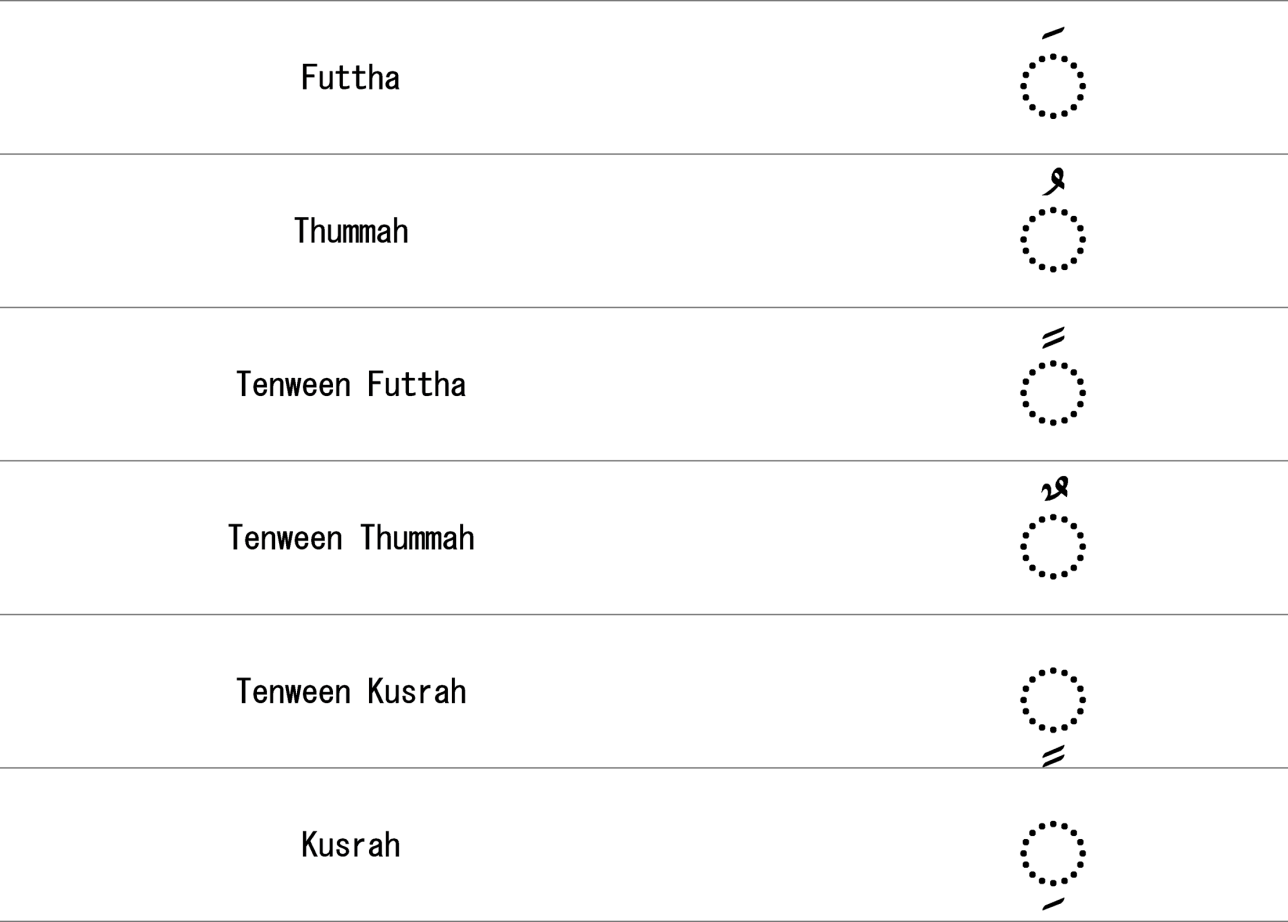
Main Arabic diacritics [18].

**Fig 3:**
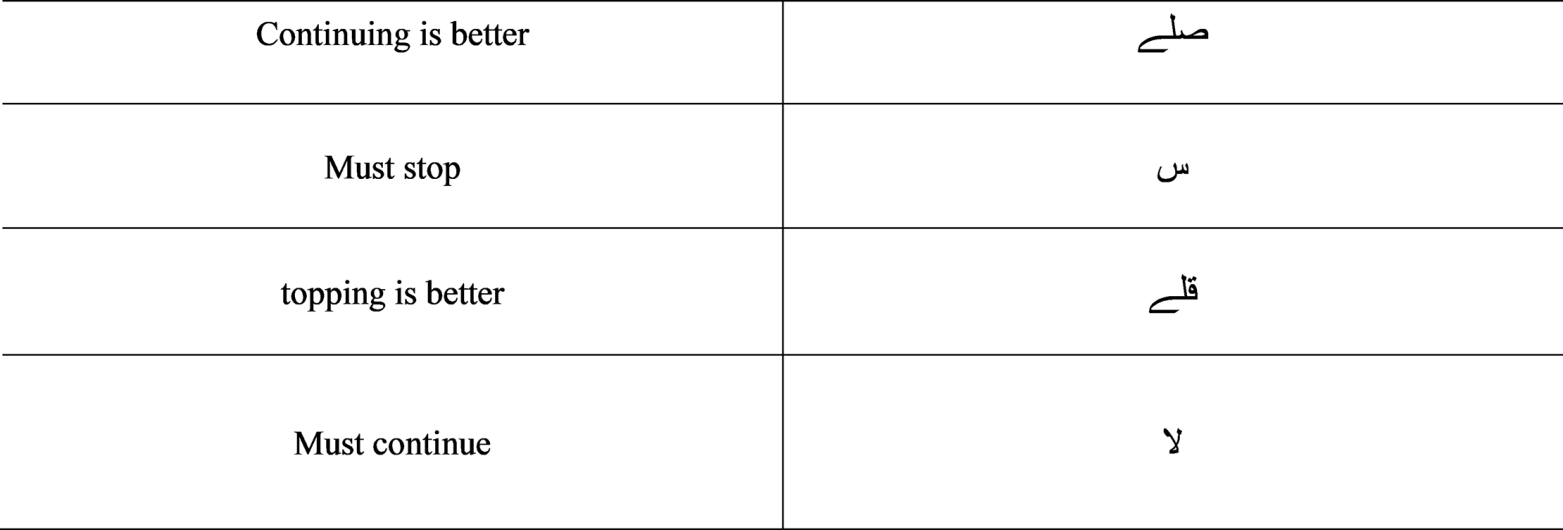
Tajweed symbols.

As shown in Fig 1, all the styles shown differ in the way diacritics and other written properties, like dots, are arranged. Most of the native speakers of Arabic do not need diacritics to read Holy Quran, as shown in Fig 1 (D) [9]. However, it is critical for non-native speakers to use these diacritics in order to recite and understand it properly [10–12]. For example, the basic diacritics of the Quran are shown in Fig 2. If the diacritics are misplaced in a verse, the whole meaning of the verse is altered [2, 10, 13]. However, most of the existing approaches related to the authentication of Digital Holy Quran (DHQ) texts remove such diacritics to improve retrieval results [14–17]. A list of the diacritic symbols and Tajweed symbols (the set of rules related to the recitation) indicate where to stop recitation and are shown in Figs 2 and 3, respectively.

**Fig 4:**
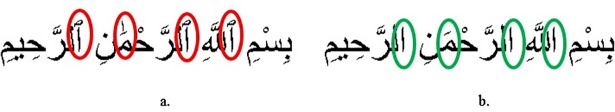
(a) Uthmanic style (b) Plain writing style verse.

Alshareef et al. [18] have proposed the Qur’an Quote verification algorithm which removes all diacritics from the input verse and authenticates the verse using a diacritic free dataset. Similarly, Yasser M. Alginahi et al. [19] has proposed an algorithm for verifying Qur’anic verses online. The approach ignores diacritics and *tashkeel* (vowel marks) for efficient verification. It converts bits of text to UTF format and authenticates through a UTF database. Alsmadi et al.[1] have used a hashing approach for authenticating Qur’anic verses without removing the diacritics. Similarly, Khalil et al. [20] and Kurniawan et al. [21] proposed the watermarking based methods to authenticate Quranic images. Most of the previous studies have focused on authenticating one single writing style. However, all these approaches are prone to fail as soon as they have to deal with different styles. Such approaches only work when the input verse and the database contain the same style. Adding or deleting one single symbol results either in a different meaning of the entire verse or causes authentication issues. One example to illustrate the difference between *Uthmani* and plain text is shown in Fig 4 where the differences are marked by red and green colour ovals.

It is observed from the *Uthmani* style, the letter *alif* 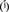 encircled with a red circle is written differently compared to the plain style. It is also noted that ‘*alif*’ written in plain style is simple and in a standard form 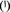. In general, in case of the *Uthmani* verses, a small *alif* 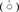 appears over the letter *mim* to express the sound of the letter while the plain script does not include it. However, both verses written in *Uthmani* and plain script are correct. Since there are no existing algorithms that can authenticate different Qur’anic writing styles from a common ground truth (dataset created and verified manually), a new approach is required that can authenticate different styles using one common database.

From the above discussion, it is clear that at present there exists no effective approach to authenticate different styles of Qur’anic texts using one common database. Hence, the focus of this paper is to propose an approach that can solve the authentication issues of Qur’anic texts available online which are written in different styles.

The structure of the paper is as follows. Section II describes the methodology, Section III presents experimental results to validate the proposed approach, and conclusions and discussions are given Section IV.

## II. Proposed approach

**Fig 5:**
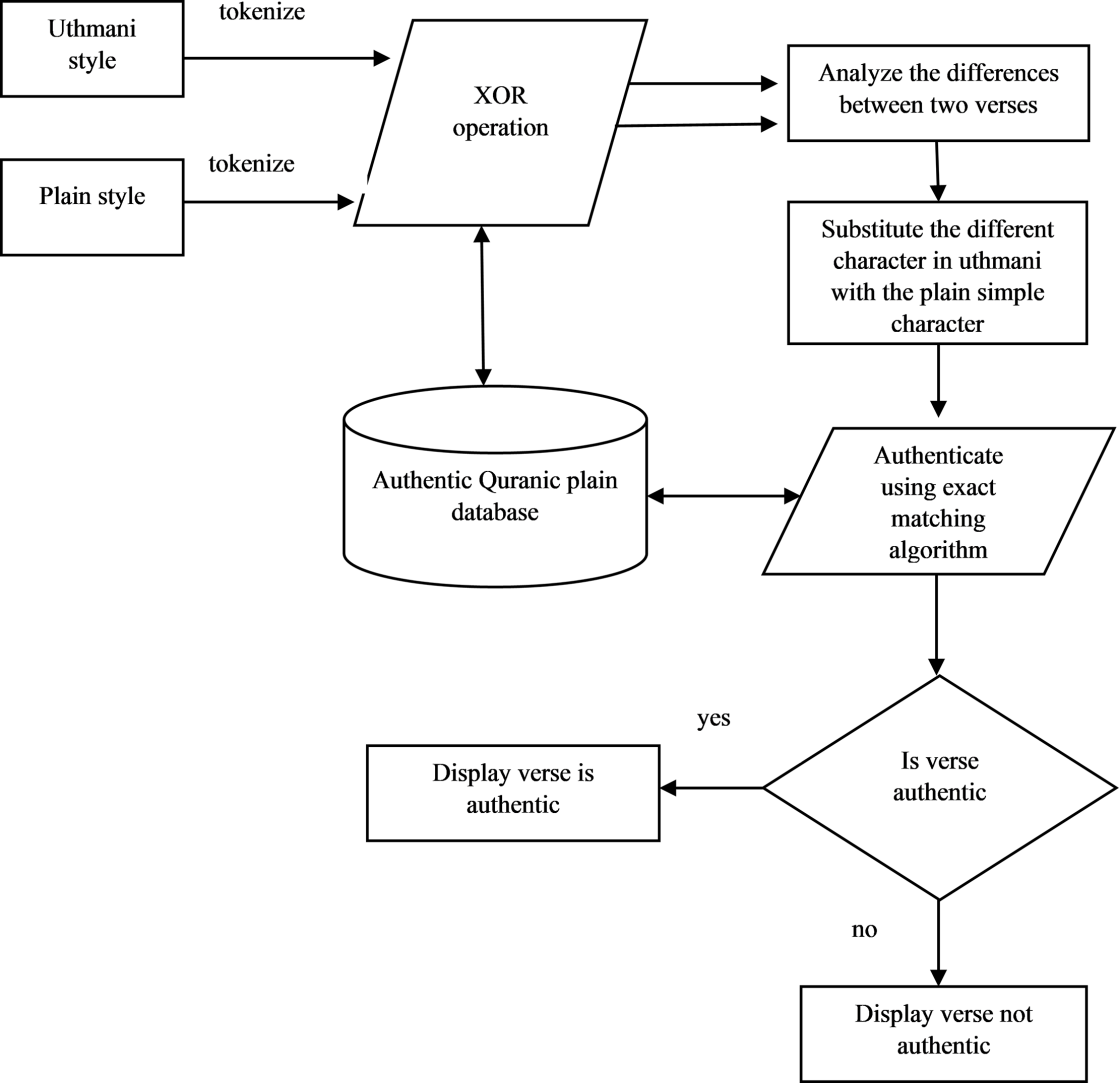
The logical flow of the proposed approach.

In this work, we consider authenticating *Uthmani* and plain Qur’an writing styles since both are widely used for communication through web or email. For each verse in the *Uthmani* style, the proposed method finds the residual by performing an XOR operation at bit level with the ground truth. The residual has been studied to find a suitable letter to substitute such that the given *Uthmani* verses can be converted to plain Qur’an text. The BMT S1 Algorithm [22] is applied to authenticate the converted *Uthmani* verse. The flow of the proposed method is shown Fig 5.

The proposed approach is divided into four sub-sections. Firstly, *Tokenization* of both the verses to segment components proposed in section (i). The residual is found using XOR operation in section (ii); the conversion is done by substituting suitable symbol with the help of ground truth in section (iii); the converted verse is verified by the S1 Algorithm in section (iv).

### (i) Tokenization for segmenting components from verse

**Fig 6:**
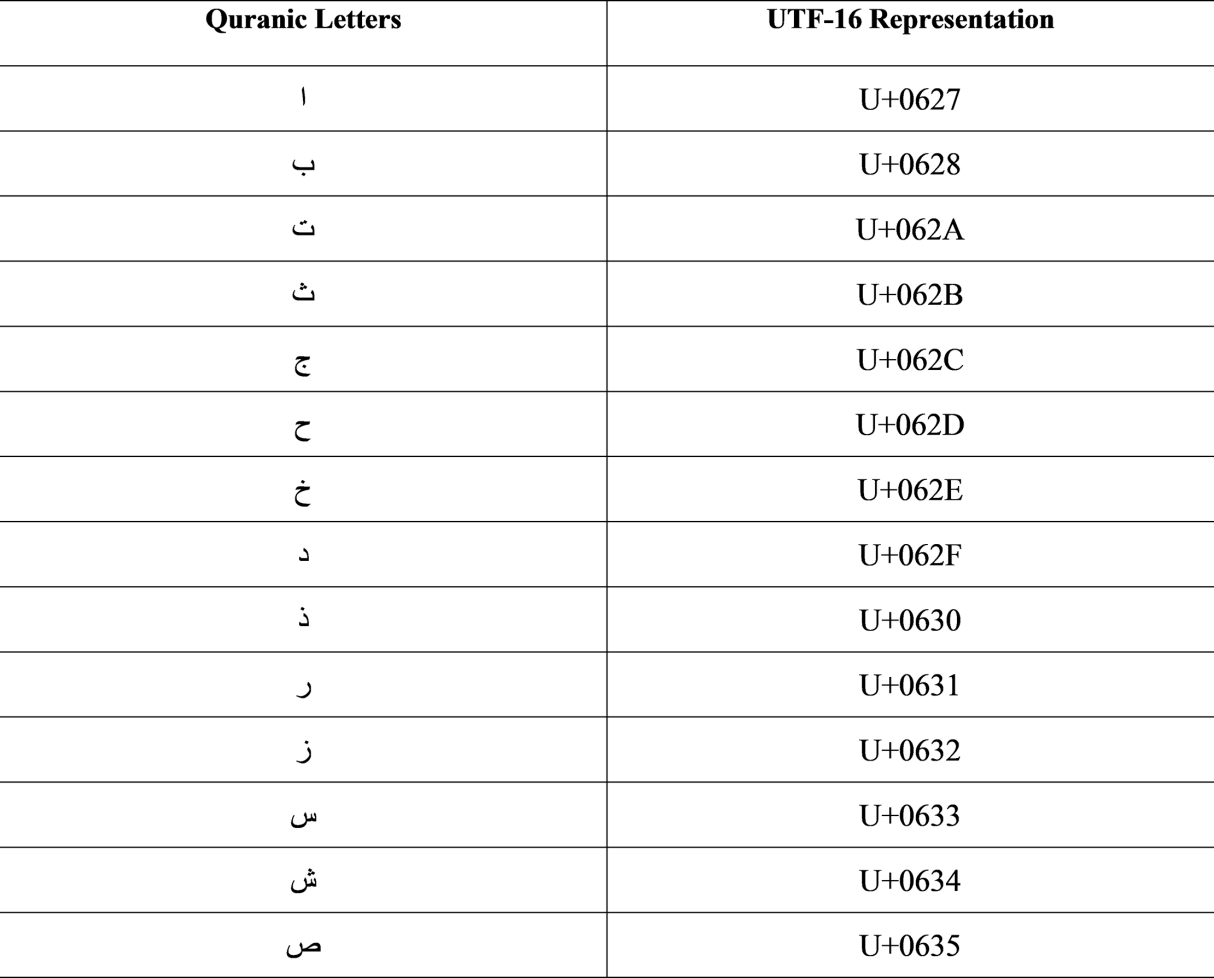
Sample UNICODE representation.

The most widely used encoding scheme for English texts is the American Standard Code for Information Interchange (ASCII). This encoding uses seven bits to represent a single English alphabet [23] which suffices for simple scripts like English, yet for complex scripts like Arabic, it is not suitable as it requires more than seven bits for representation. Therefore, to handle complex text, generally, the UTF 16 encoding scheme is used because UTF 16 constitutes a variable length encoding [23] scheme which represents Arabic text with diacritical symbols considerably well. Samples of the Unicode for Arabic letters are shown in Fig 6.

**Fig 7:**
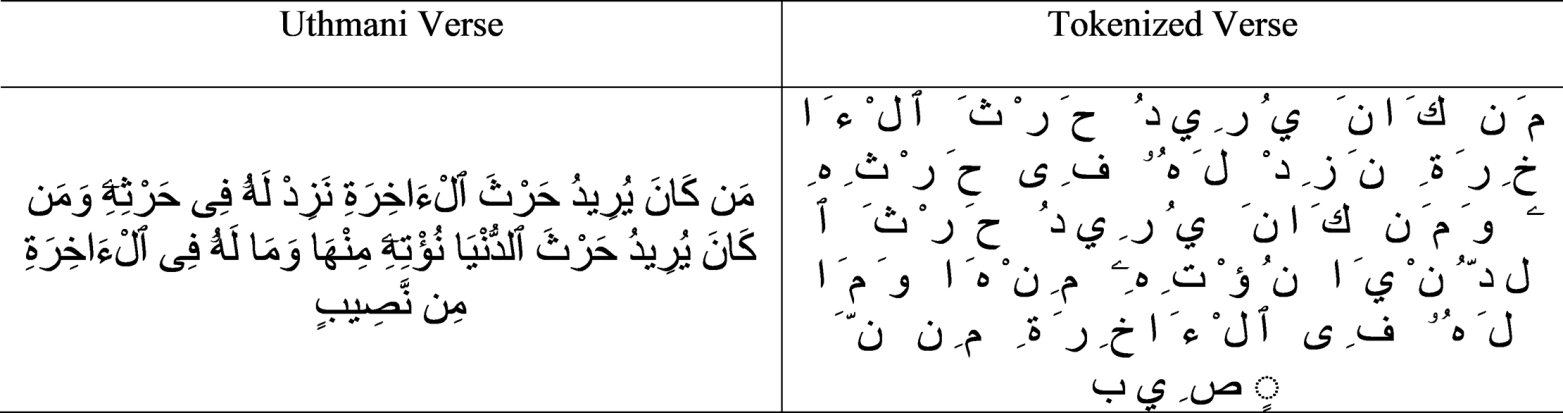
Tokenized quranic verse.

The proposed approach uses Unicode of Arabic text for segmenting components (characters) from a given verse. In this research, we propose to explore the regular expression approach [24, 25] for splitting verse into character components as it provides the delimiter (“”) which splits a given string into character by character with the help of Unicode. For more details for segmenting character component from verse can be found in [25]. An example of character component segmentation is shown for the Uthmani verse in Fig 7.

### (ii) XOR operation for residual

The bits segmented character components of the *Uthmani* text are compared with the bits of ground truth character components by performing the XOR operation whose outputs are true if both inputs are correct [26]. If this is not the case, the difference is called ‘residual’. For every input of the *Uthmani* text, the proposed approach finds ground truth created by plain text. If both verses are correct, the XOR operation outputs 0 else 1 as shown in Table 1 where one can see “1” marked in bold representing the residual of the *Uthmani* and the plain Qur’anic text.

**Table 1:**
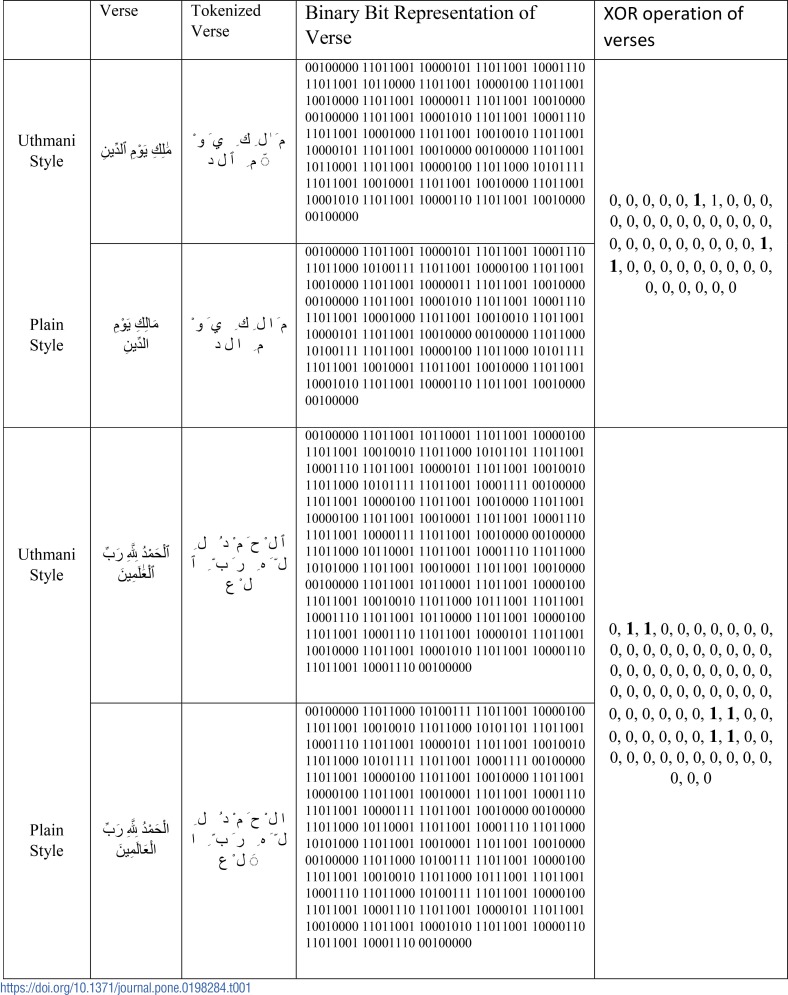
XOR operation of verses.

As presented in Table 1, the number of 1’s highlighted in red depict the differences between two strings. Finally, all major differences between the two writing styles are analyzed using the proposed approach. The analyzed results are retrieved using the dynamic programming approach [27] and placed in the substitution phase.

### (iii) Substitution for correction

In order to correct the difference given by the previous step and convert the *Uthmani* style into plain style, symbols are created manually after analyzing the differences between *Uthmani* and plain Qur’anic styles as shown in Table 2. The proposed approach finds the difference and then identifies the suitable symbol to substitute the residual in order to restore the meaning of the *Uthmani* characters.

**Table 2:**
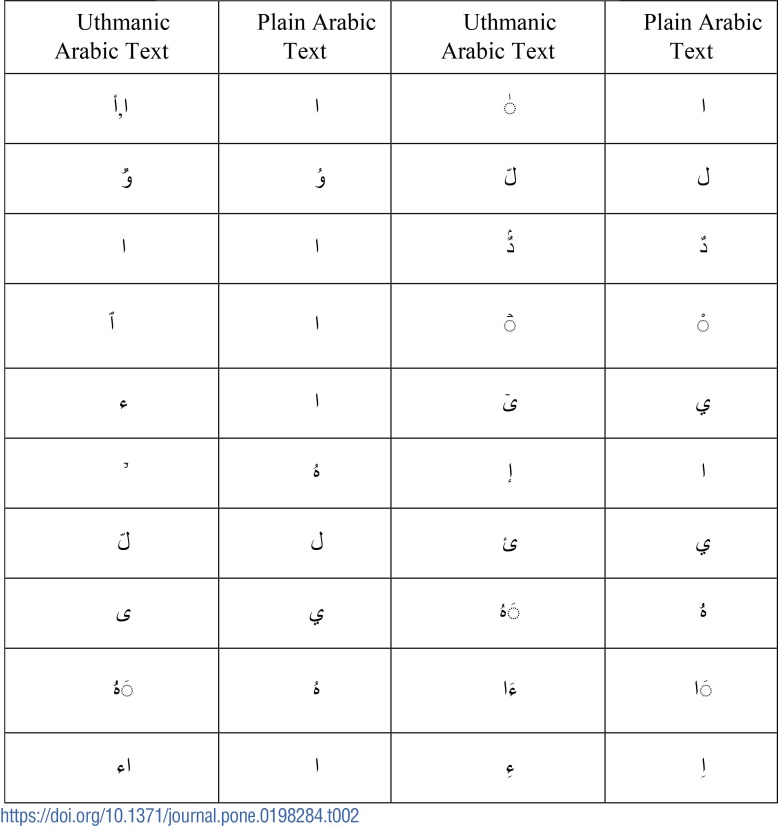
Analysis of Uthmanic and plain quranic verses.

For instance, the changes made in Table 2 include the replacement of letters like different versions of the letter *alif* 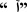, “
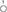
(Arabic subscript alef) ” with a simpler one i.e. 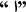. Similarly, letters like 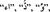 were replaced by their simpler forms as shown in Table 2. The symbol 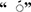 (*shadda*) is used to represent one letter twice (long consonant) during recitation [28]. Similarly, the symbol 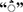 (Arabic small high dotless head of 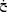
is replaced by 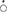
(sukoon)). The purpose of placing a *sukoon* above or beneath the letter is to indicate no sound, while a dotless head of 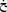
signifies the absence of a vowel. Some styles use sukoon, while some use the dotless 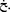 Hence, for improving the accuracy of authentication, the symbols including 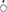 and 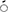
were removed. Similarly, all forms of the letter *yaa* 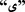 are substituted with a simpler form, i.e. 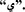 The other symbols that are removed to improve the detection accuracy are listed in Table 3. Removing these symbols does not alter the meaning [18].

**Table 3:**
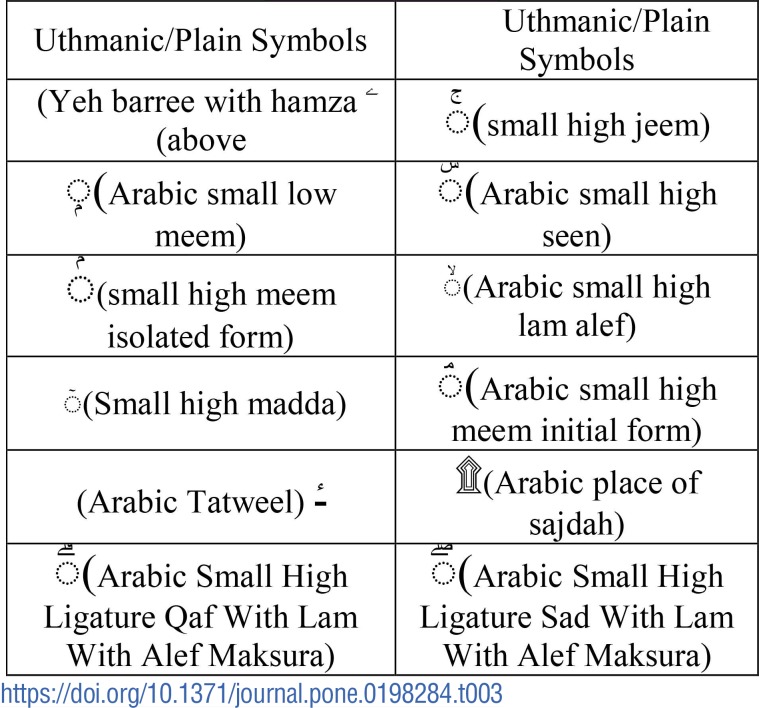
Symbols removed.

Samples of the symbols used for substitution are listed in Table 4.

**Table 4:**
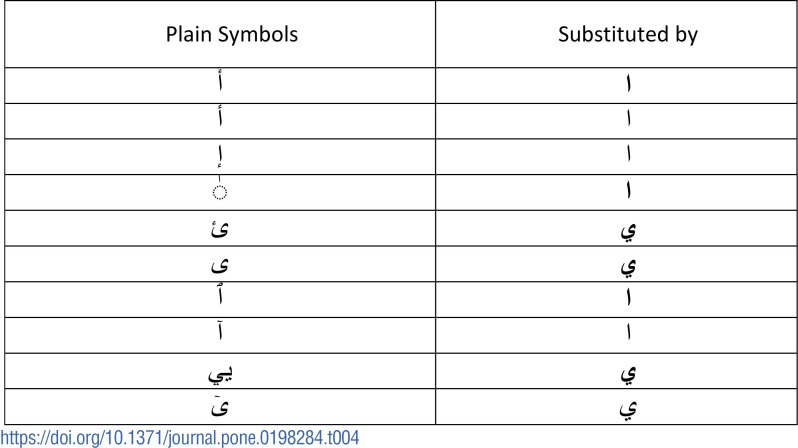
Pre-processing in benchmark dataset.

### (iv) Exact matching for authentication

In order to validate the incorporated correction done in the previous step, we propose to use the exact matching algorithms. For choosing an optimal exact matching algorithm, we analyzed the performance of different character-based exact matching algorithms using data-sets from tanzil.net as shown in Table 5.

**Table 5:**
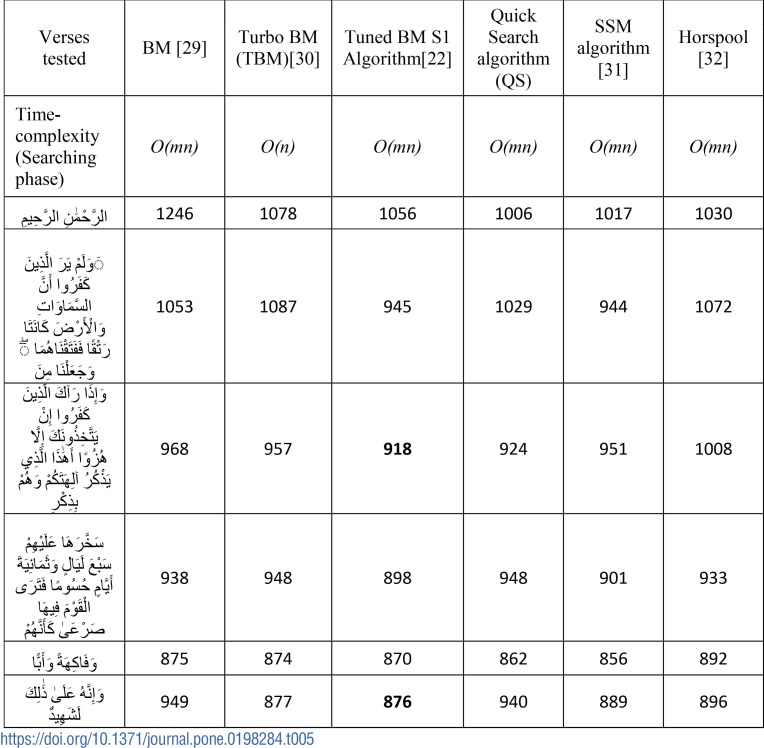
Performance analysis of character-based exact matching algorithms.

From experiments, it was observed that there is no clear winner from different variants of Boyer-Moore’s character-based algorithms. Different tested algorithms included Boyer-Moore algorithm [29], turbo Boyer-Moore algorithm[30], tuned boyer Moore algorithm[22], horspool algorithm [32] and SSM algorithm [31]. It can be observed S1 Algorithm in Table 5 performed slightly better compared to other approaches. Hence, S1 Algorithm was applied for matching purpose.

**Fig 8:**
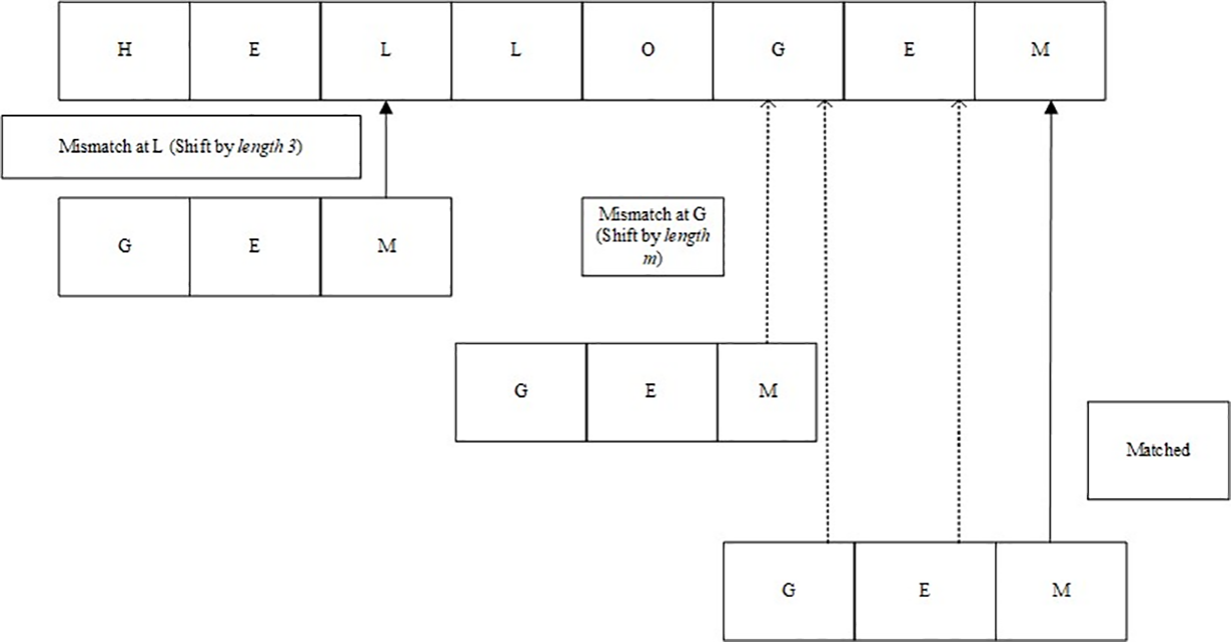
Boyer Moore algorithm.

However, in order to understand the methodology of above-tested algorithms including S1 Algorithm, it is must to have a good understanding of Boyer-Moore (BM) algorithm. Boyer-Moore algorithm [29, 33] starts searching characters from right to left of the given pattern. In case of a mismatch, it shifts as many as *m* characters as shown in Fig 8. *(here m denotes the length of pattern to be searched and n denotes the length of given text)*.

The algorithmic steps in BM are as follows.

1. Searching for a given pattern from the right side of the window and using the bad match table to skip characters in case of a mismatch.
  - Pre-processing: In this stage, a table is created which gives values regarding how much shift is required in case of a mismatch (bad-match table). Once a character mismatch occurs, the algorithm shifts to the right side of the pattern according to the value given in the bad-match table.
  - Searching starts from the tail of the pattern, i.e. from the right to left side of the text as compared to the naive algorithm where searching starts from the left. The algorithm works by computing the length of the search string and storing its value as default shift length.
  - The values can be computed using *Value = Length of pattern-1-index of character*

S1 Algorithm, on the other hand, is a simpler version of BM. The difference between BM and this algorithm is that it takes longer shifts and scans the text segment till that segment is a suffix of the pattern. This algorithm remembers the suffix of last matched substring of the pattern due to which it is possible to jump over that sub-string and allows execution of turbo-jump, which is a memory match.

### (v) Algorithm description

In the whole process of authentication, there are two phases as mentioned in section II. The description of the whole authentication process is given in the form of algorithmic steps shown below:

***Begin***

***1*. *Pre-processing phase*:**

    *(i) Tokenisation Phase*

    *Input = uthmani(u) and plain quranic verse(nu)*

        *int uL = u*. *Length*

        *int nuL = nu*. *Length*

        *For i = 0 to uL-1 && nuL-1*

            *String [] s1 = tokenised u*

            *String [] s2 = tokenised nu*

    ***(ii) XOR Operation phase***

        *For i = 0 to 11*. *length-1 && s2*. *length-1*

            *Output[i] = s1^s2 (XOR operation)*.

        *If (output[i] = = 0)*

            *Display = Output[i]*

            *i++*

        *else*

            *residual[i] = different characters in s1*

        *For i = 0 to s1*. *length-1*

            *Substitute[i] = Replace residual[i] with the value from manually analysed table*.

        *Repeat XOR operation phase*.

        ***End***

        ***2*.*Searching phase*:**

        *Begin*

        *For i = 0 to display*. *length*

        *Search the given verse using* **S1** *Algorithm*

        *End*

In the pre-processing phase, the length of uthmani and plain quranic verse is calculated and stored in the variables *u* and *nu*. Both the verses are tokenized using regular expression approach (delimiter method) controlled by *for* loop. The tokenized verses are stored in their respective variables of type *String*(s). Since both the strings are in the tokenized form, it becomes easy to identify the differences between the two verses. The result of XOR operation between the two verses is stored in a variable *display*. In case, the value of *output[i]* comes out to be zero, there is no need for substitution. However, in case the *output[i]* is other than zero, then that particular verse is analyzed again to check which character is different than the other benchmark verse (plain Quranic style). Finally, the different character is substituted from the simpler version analysed in Table 2. In the end, searching phase starts, where *S1* Algorithm uses *for* loop to iterate through all the characters of the input tokenized substituted output and compare with the database for authenticity.

### vi. Complexity study

The time complexity of the proposed approach can be divided into two parts: pre-processing phase and searching. Let *u* and *nu* be the two verses to be converted into a single format as explained above. For tokenization phase, to segment both verses, the time taken will be linear in the worst case with *n* representing the size of the text. Hence, for tokenization phase, complexity will be *O(n)*.

Since XOR operation, again requires *n* characters to be processed and requires substitution based on XOR difference, complexity for XOR phase will be O(n)+O(n*(size of the table)).

However, as *n* increases, the size of the table becomes irrelevant and the time complexity in the worst case will be *O(n)*. Here table represents values that were analyzed based on differences between Uthmani and plain Arabic texts. Finally searching process needs O(p*t) time (i.e. linear time), where *p* represents the pattern to be searched after pre-processing phase and *t* represents the given text in the worst case. Hence, the total time complexity of the proposed approach for the phases of tokenization, XOR operation and the search process is *O(n)+ O(n)+ O(n)*. In other words, total time complexity is *O*(n)(1+1+1), as *n* becomes larger, constants can be ignored resulting in time-complexity of O(n).

## III. Experimental results

In order to evaluate the proposed approach, we consider the standard and authentic version of a Qur’an dataset available in Tanzil.net which has been used in previous research. This dataset was further verified by experts. Tanzil.net has six types which include *Uthmani*, *Simple*, *Simple Enhanced*, *Simple Minimal*, *Simple Clean and Uthmani Minimal* [8]. In this work, we consider a *Simple* dataset as it contains all diacritics that are necessary to recite the Qur’anic text accurately. Besides, it consists of fewer symbols which reduce the number of computations for verse verification. The pre-processed and verified S1 Dataset (by the Faculty of Islamic Studies, University of Malaya) is available on the website (http://quranhadith.fsktm.um.edu.my/).

### (i). Experiments for authentication

**Fig 9:**
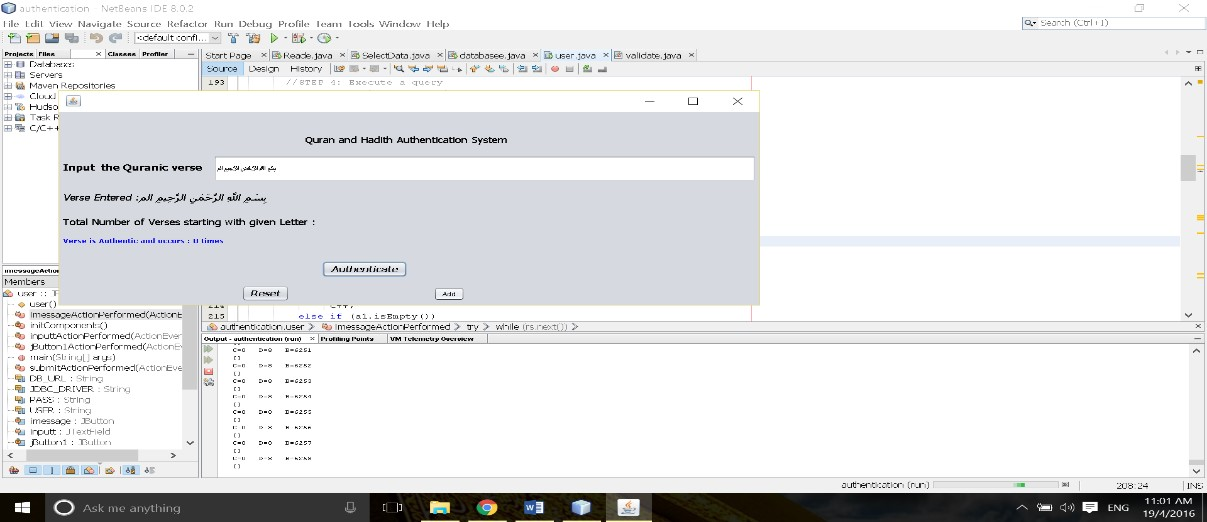
Prototype.

The prototype of the proposed approach is shown in Fig 9 which illustrates how the proposed method finds residual and the correct verse. The system details for conducting experiments include Java with IDE Netbeans 8.02. The hardware used includes an i-5 Intel Processor 4 MB cache and a 4 GB RAM with a Windows 10 Operating system. We randomly choose 1000 Qur’anic *Uthmani* verses from the database in order to measure the performance.

The proposed approach authenticated 871 verses out of 1000 verses of the Digital Qur’an. The experiments were done on small, medium and long chapters of Digital Quran.

$$Accuracy=\frac{Number\;of\;particular\;verses\;Found}{Total\;number\;of\;particular\;verses}$$

Thus, Accuracy = 871/1000 = 87.1%.

### (ii). The effectiveness of the proposed approach

In order to show the usefulness of the proposed approach which converts the verse by substituting suitable symbols at their residual locations, we conduct experiments by feeding input directly to S1 Algorithm and authenticate without correction as shown Table 6. Basically, the algorithm checks whether the *Uthmani* verses can be authenticated in the *Plain* Qur’an dataset. As shown in Table 6, the S1 Algorithm fails to detect the verses due to a different arrangement of diacritics in the *Uthmani* and the *Plain* dataset. However, when the corrected verse given by the proposed approach for the S1 Algorithm is fed, the same verses shown in Table 6 are authenticated correctly. Thus, the proposed conversion by substitution proves useful and effective.

**Table 6:**
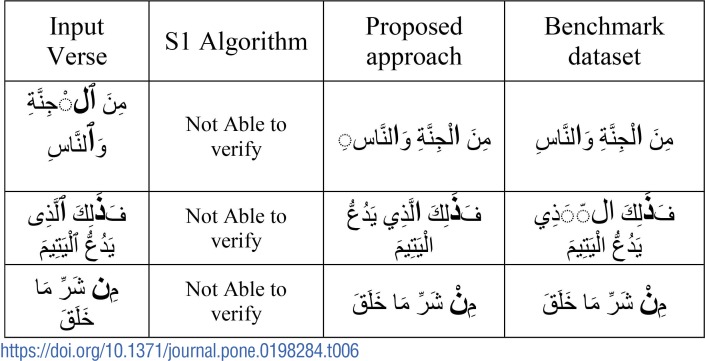
Analysis without using XOR and substitution.

**Fig 10:**
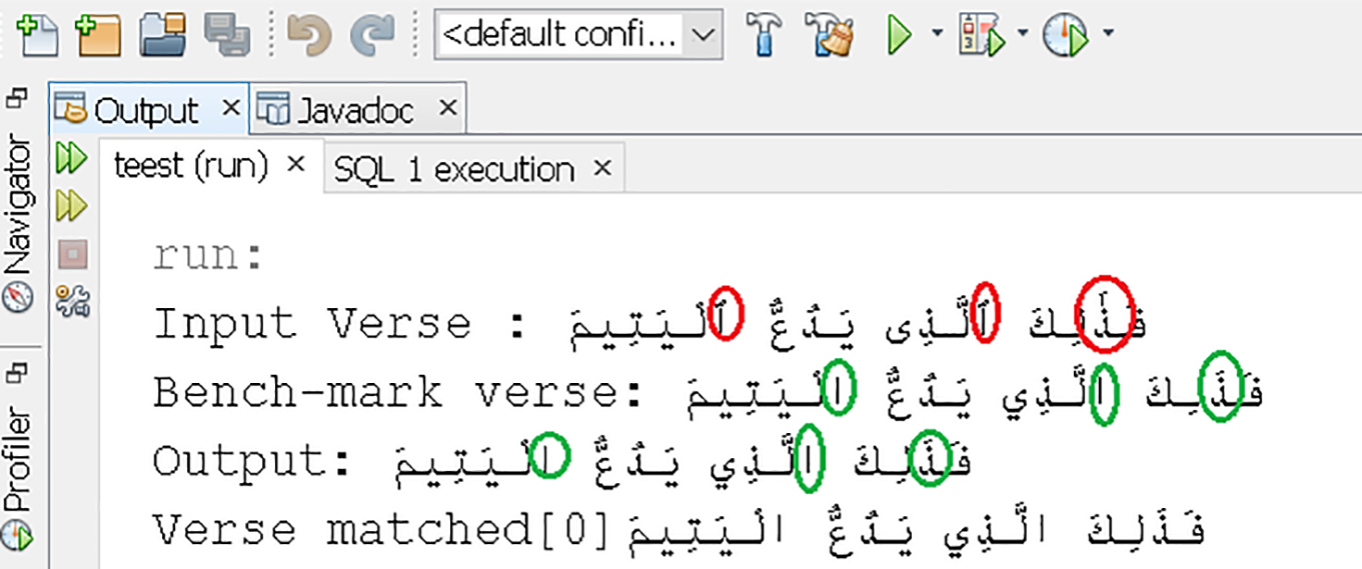
Prototype snapshot.

The snap shot of experiments performed shown in Table 6 is given in Fig 10.

### (iii). Comparative study

In order to show the superiority of the proposed approach, we compare its results with the other existing approaches. We consider the Qur’an Quote Verification Algorithm (QQV) which removes all diacritics from the input Qur’anic verse and verify the authenticity by using the data-set [18]. Qur’an Verification and Authentication Algorithm which encodes input using the UTF encoding scheme and verify it using the UTF-based dataset [19] and the Hashing Algorithm which generates a hash using existing algorithms like MD5. Then the authenticity is verified based on the hash values from the given dataset [1]. The sampled qualitative and quantitative results of the proposed approach and the other existing approaches are shown in Table 7 where all existing approaches fail to authenticate due to a mismatch between the *Uthmanic* verse input and the *Plain* Qur’an verse. This is valid because both verses differ in their arrangement of the diacritics. Therefore, the accuracy of authentication of the existing approaches is 0.0% while the proposed approach method achieves 87.1% accuracy. It corrects the mismatch between the *Uthmani* verse and the *Plain* Qur’an verse through residual finding and substitution as shown in Table 7.

**Table 7:**
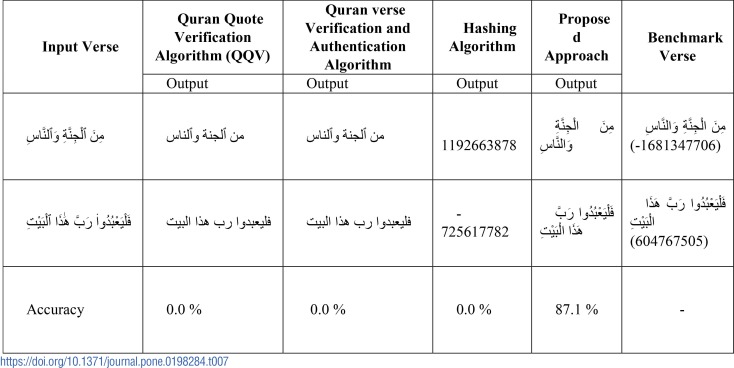
Comparative analysis after XOR and substitution phase.

Since we consider the text in *Simple Uthmani* using the simple and common *alif*, the proposed approach does not work well for the verses which contain extra characters. For example, the following Uthmanic verse 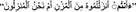 starts with a letter 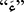 and the plain verse 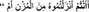

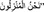
 starts with a letter 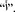. Since both the verses are correct, but there is no substitution possible for these kinds of verses. In case, a letter 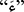 is substituted with the letter 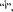 then the remaining verses of Digital Quran containing a letter 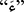 will also change resulting in a more severe problem. Similarly, the following plain verse 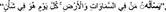 contains extra alif in word 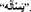 In Uthmanic version, the word 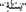 does not contain any extra alif 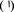 and the letters 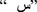 and 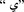 are connected directly. This results in a mismatch. Those types of words result in lower accuracy. A few other samples for which the proposed approach does not perform well are listed in Table 8. From Table 8, it can be observed, there are cases (for example serial no. 1,2 6,7), where some extra characters like *alif* are embedded in Uthmanic text compared to reference database making conversion process inevitable. Similarly, in serial no. 8, the character 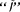 in plain text style is represented by 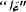 in Uthmanic style. In this kind of cases, substitution method is not feasible considering sensitive nature of Quran. Therefore, there is scope for extension of the proposed work in order to find a solution to the above-mentioned issue.

**Table 8:**
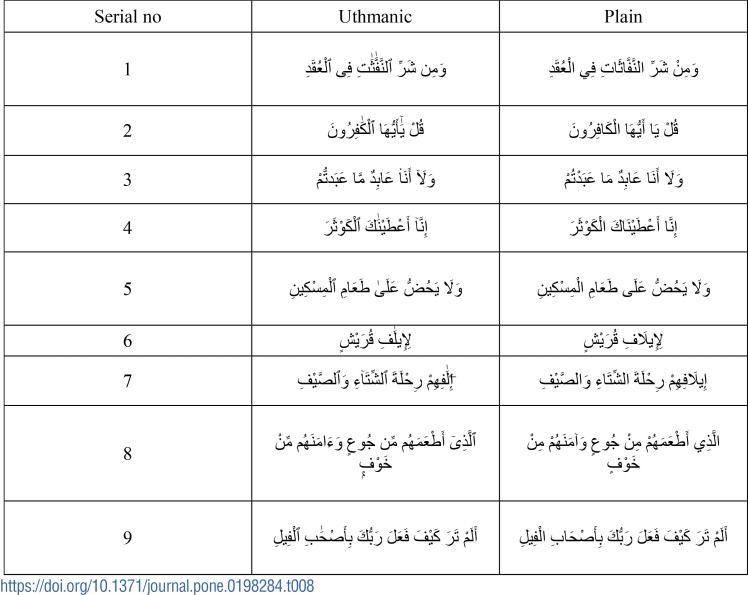
Unverified verses.

## IV. Conclusion

This paper has proposed a new approach for authenticating the Qur’anic verses written in different styles using one single database. The proposed approach finds residual between the input *uthmani* verse and the *Plain*text by performing the XOR operation. The proposed approach studies the residual to find a suitable symbol and substitute the error symbol (*Uthmani* letters differing from the *Plain* style). Furthermore, the corrected version (converted *Uthmani*) has been validated through the S1 Algorithm. The experimental results show that the proposed approach achieves 87.1% accuracy for authentication. In addition, the proposed approach outperforms the existing approaches in terms of accuracy. The existing approaches do not perform well as they are only suitable for single types of writing styles.

Our future work will focus on enhancing the verification phase by working on the limitations of the proposed approach and extending it to solve more complex styles. There are still lot of issues that need to be addressed. Firstly, the accuracy of the proposed approach need to improve. Secondly, the availability of digital Quran in different styles is other pressing research problem. It would be interesting to extend the proposed approach to authenticate other styles as well. Besides, it will be interesting to work on improving the time complexity of our proposed approach and evaluating its accuracy on large datasets. Moreover, our immediate goal is to make this web-based system publicly available and extend the platform for android based Quran authentication system for mobile phone users.

## Supporting information

S1 Dataset Al-Quran dataset. (TXT)

S1 Algorithm BMT algorithm. (RTF)
