# Supplementary material for: Residual-based approach for authenticating pattern of multi-style diacritical Arabic texts
Source: PLoS One. 2018 Jun 20;13(6):e0198284. doi: 10.1371/journal.pone.0198284 (PMC6010264; doi:10.1371/journal.pone.0198284)
Supplement: S1 Algorithm — (RTF) [file pone.0198284.s002.rtf]

package com.javacodegeeks.stringsearch;

import java.util.ArrayList;
import java.util.List;

public class BMT {

	private static void preBmBc(char[] x, int bmBc[]) {
		int i, m = x.length;

		for (i = 0; i < bmBc.length; ++i)
			bmBc[i] = m;
		for (i = 0; i < m - 1; ++i)
			bmBc[x[i]] = m - i - 1;
	}

	private static int arrayCmp(char[] a, int aIdx, char[] b, int bIdx,
			int length) {
		int i = 0;

		for (i = 0; i < length && aIdx + i < a.length && bIdx + i < b.length; i++) {
			if (a[aIdx + i] == b[bIdx + i])
				;
			else if (a[aIdx + i] > b[bIdx + i])
				return 1;
			else
				return 2;
		}

		if (i < length)
			if (a.length - aIdx == b.length - bIdx)
				return 0;
			else if (a.length - aIdx > b.length - bIdx)
				return 1;
			else
				return 2;
		else
			return 0;
	}

	public static List<Integer> findAll(String pattern, String source) {
		char[] x = pattern.toCharArray(), src = source.toCharArray();
		char[] y = new char[src.length + x.length];
		System.arraycopy(src, 0, y, 0, src.length);
		int j, k, shift, m = x.length, n = src.length;
		List<Integer> result = new ArrayList<Integer>();

		int[] bmBc = new int[65536];

		/* Preprocessing */
		preBmBc(x, bmBc);
		shift = bmBc[x[m - 1]];
		bmBc[x[m - 1]] = 0;
		for(int i = 0; i < m; i++)
			y[n + i] = x[m - 1];

		/* Searching */
		j = 0;
		while (j < n) {
			k = bmBc[y[j + m - 1]];
			while (k != 0) {
				j += k;
				k = bmBc[y[j + m - 1]];
				j += k;
				k = bmBc[y[j + m - 1]];
				j += k;
				k = bmBc[y[j + m - 1]];
			}
			if (arrayCmp(x, 0, y, j, (m - 1)) == 0 && j + m - 1 < n)
				result.add(j);
			j += shift; /* shift */
		}

		return result;
	}
	
	public static BMT compile(String pattern) {
		char[] x = pattern.toCharArray();
		int shift, m = x.length;

		int[] bmBc = new int[65536];

		preBmBc(x, bmBc);
		shift = bmBc[x[m - 1]];
		bmBc[x[m - 1]] = 0;
		
		BMT bmt = new BMT();
		bmt.bmBc = bmBc;
		bmt.m = m;
		bmt.shift = shift;
		bmt.x = x;
		
		return bmt;
		
	}
	
	public List<Integer> findAll(String source) {
		char[] src = source.toCharArray();
		char[] y = new char[src.length + x.length];
		System.arraycopy(src, 0, y, 0, src.length);
		int j, k, n = src.length;
		List<Integer> result = new ArrayList<Integer>();
		
		for(int i = 0; i < m; i++)
			y[n + i] = x[m - 1];
		
		j = 0;
		while (j < n) {
			k = bmBc[y[j + m - 1]];
			while (k != 0) {
				j += k;
				k = bmBc[y[j + m - 1]];
				j += k;
				k = bmBc[y[j + m - 1]];
				j += k;
				k = bmBc[y[j + m - 1]];
			}
			if (arrayCmp(x, 0, y, j, (m - 1)) == 0 && j + m - 1 < n)
				result.add(j);
			j += shift; /* shift */
		}

		return result;
	}
	
	private char[] x;
	private int shift, m;
	private int[] bmBc;

}
